# Multiple cone pathways are involved in photic regulation of retinal dopamine

**DOI:** 10.1038/srep28916

**Published:** 2016-06-30

**Authors:** Sheng-Nan Qiao, Zhijing Zhang, Christophe P. Ribelayga, Yong-Mei Zhong, Dao-Qi Zhang

**Affiliations:** 1 Institutes of Brain Science, Fudan University, Shanghai 200032, China; 2 Eye Research Institute, Oakland University, Rochester, MI 48309, USA; 3 Ruiz Department of Ophthalmology and Visual Science, McGovern Medical School, The University of Texas Health Science Center at Houston, Houston, TX 77030, USA; 4 Graduate School of Biomedical Sciences, The University of Texas Health Science Center at Houston, Houston, TX 77030, USA; 5 State Key Laboratory of Medical Neurobiology, Fudan University, Shanghai 200032, China; 6 Collaborative Innovation Center for Brain Science, Fudan University, Shanghai 200032, China

## Abstract

Dopamine is a key neurotransmitter in the retina and plays a central role in the light adaptive processes of the visual system. The sole source of retinal dopamine is dopaminergic amacrine cells (DACs). We and others have previously demonstrated that DACs are activated by rods, cones, and intrinsically photosensitive retinal ganglion cells (ipRGCs) upon illumination. However, it is still not clear how each class of photosensitive cells generates light responses in DACs. We genetically isolated cone function in mice to specifically examine the cone-mediated responses of DACs and their neural pathways. In addition to the reported excitatory input to DACs from light-increment (ON) bipolar cells, we found that cones alternatively signal to DACs via a retrograde signalling pathway from ipRGCs. Cones also produce ON and light-decrement (OFF) inhibitory responses in DACs, which are mediated by other amacrine cells, likely driven by type 1 and type 2/3a OFF bipolar cells, respectively. Dye injections indicated that DACs had similar morphological profiles with or without ON/OFF inhibition. Our data demonstrate that cones utilize specific parallel excitatory and inhibitory circuits to modulate DAC activity and efficiently regulate dopamine release and the light-adaptive state of the retina.

Dopamine acts as a neurotransmitter in the CNS and plays an important role in a variety of brain functions such as movement, memory, pleasure, reward, and cognition. In the neural retina, dopamine can influence rod vision^1^, mediate light adaptation of the retina^2^,^3^,^4^,^5^,^6^, reset the phase of the retinal biological clock^7^,^8^, and suppress melatonin release^9^,^10^,^11^. Retinal dopamine is synthesized and released from dopaminergic amacrine cells (DACs), and the release of dopamine from these cells is greatly enhanced by light^12^,^13^. However, investigations of the cellular and synaptic mechanisms by which light regulates DAC activity have just begun^14^,^15^,^16^,^17^,^18^. Initial studies have demonstrated that light can increase DAC activity by activating all types of photosensitive retinal cells—the classical rod and cone photoreceptors and also the recently discovered melanopsin-expressing intrinsically photosensitive retinal ganglion cells (ipRGCs)^14^,^15^,^17^,^18^.

Cones are known to use parallel ON and OFF pathways that provide information about increases and decreases in light levels, respectively. The cones transmit their signals through bipolar cells to amacrine and ganglion cells. The bipolar cells initiate the ON and OFF pathways. ON bipolar cells carry a metabotropic glutamate receptor, mGluR6, that inverts the sign of the cone signal^19^, whereas OFF bipolar cells express ionotropic glutamate receptors of the kainate/(RS)-α-amino-3-hydroxy-5-methyl-4-isoxazolepropionic acid (AMPA) type, which preserve the sign of the cone signal^20^,^21^. Anatomically, ON bipolar cells ramify in the inner part (sublamina b) of the inner plexiform layer (IPL), where they provide excitatory responses to amacrine and ganglion cells at light onset (ON responses). In contrast, OFF bipolar cells terminate in the outer part (sublamina a) of the IPL, where they provide excitatory responses to amacrine and ganglion cells at light offset (OFF responses).

DACs have processes that are highly stratified in sublamina a and they are presumed to possess excitatory OFF responses. However, previous studies in the mouse retina have shown that DACs exhibit an inhibitory ON response at low light intensities and excitatory ON and inhibitory OFF responses at high light intensities^14^,^15^,^17^. It remains unclear how cones and their neural circuits contribute to inhibitory and excitatory responses of DACs. This problem is difficult to solve, because mouse cone signals cannot be simply distinguished from rod signals based on stimulation wavelength or intensity (Mouse cones do not express a long-wavelength opsin, but an opsin sensitive to middle wavelengths with a peak at 510 nm, greatly overlapping with that of the rods^22^).

It is also unknown whether cones drive DACs via ipRGCs within the retina. ipRGCs represent a small population of retinal ganglion cells that express the photopigment melanopsin and are intrinsically sensitive to light^23^,^24^. ipRGCs also receive inputs from rods and cones through ectopic ON bipolar cells in the mammalian retina^16^,^25^,^26^,^27^. Combining melanopsin-based photoresponses with rod/cone signals, ipRGCs primarily transmit integrated photic signals from the retina to a variety of brain nuclei involved in non-image-forming visual functions such as circadian photoentrainment and the pupillary light reflex^28^,^29^. However, recent studies have demonstrated that ipRGCs could also influence retinal development^30^,^31^,^32^ and regulate the physiological functions of upstream retinal neurons such as DACs^15^,^17^,^18^,^33^. Therefore, it has been hypothesized that in addition to melanopsin-based signalling, ipRGCs are capable of conveying rod/cone signals from ON bipolar cells to DACs. This hypothesis is attractive because it could provide a pathway for retrograde signal flow from the innermost retina back to the outer retina. However, this idea still lacks experimental support.

In this paper, we sought to address: (1) what kinds of light responses cones alone generate in DACs, (2) how ON and OFF bipolar cells transmit cone signals to DACs, (3) whether ipRGCs convey cone signals to DACs, (4) whether distinct light-responsive DACs have the same morphological profiles, and (5) whether cones alone are sufficient to regulate dopamine release during light adaptation.

## Results

### Cones generate three classes of light-evoked responses in DACs

To define the contribution of cones to DAC light responses, we bred double knockout mice in which rod and melanopsin function are eliminated and referred to herein as cone-function-only mice (see Materials and Methods). The loss of rod function was validated by electroretinogram (see Supplementary Fig. S1), whereas elimination of melanopsin expression was confirmed by immunocytochemistry (see Supplementary Fig. S2). In addition, it is unlikely that DACs are compromised by the loss of rod and melanopsin function because their density remained unchanged compared to wild-type mice (see Supplementary Fig. S3).

**Figure 1:**
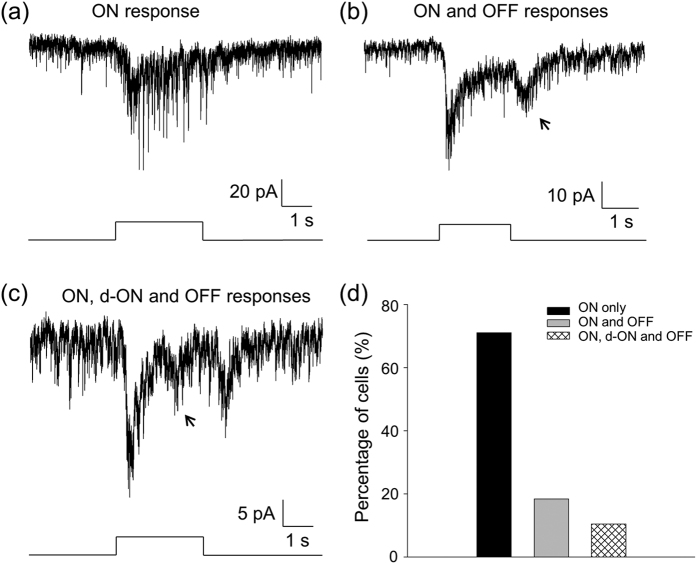
Cones generate three classes of light-evoked responses in DACs. Whole-cell voltage-clamp recordings (*V*_*hold*_ = −65.5 mV) were made on DACs in flat-mount retinas isolated from cone-function-only mice. **(a)** A typical DAC exhibited a light-evoked inward current immediately after light onset (ON response). **(b)** In contrast to the cell in (**a**), this cell had an additional light-evoked inward current at light offset (OFF response, indicated by an arrow). **(c)** A typical cell displayed an inward current at ~1 s after light onset (d-ON response, arrow) between the ON and OFF responses. Stimulation bar under each trace shows timing of a 470-nm light pulse. Light stimulus duration was 3 s for (**a**) and (**c**) and 2 s for (**b**). **(d)** Of a total of 86 cells tested, 68.6% only had ON responses (black bar), 20.9% exhibited ON and OFF responses (grey bar), and 10.5% had all three classes of light responses (striped bar).

**Figure 2:**
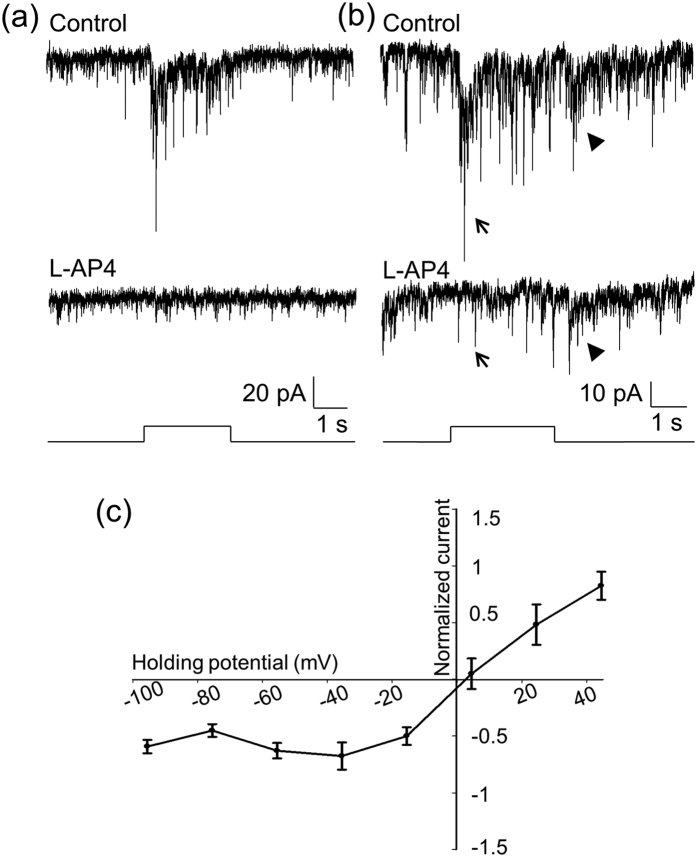
L-AP4 blocks ON responses of DACs that have an excitatory reversal potential. (**a)** The ON response of a DAC (top trace) was completely blocked by L-AP4 (bottom trace). **(b)** This cell had ON and OFF responses (top trace). The bottom trace shows that the ON response was blocked by L-AP4 (indicated by an arrow), whereas the OFF response was still persistent (indicated by an arrowhead). Stimulation bar under each trace shows timing of a 470-nm light pulse. Light stimulus duration was 2 s for (**a**) and 3 s for (**b**). **(c)** Normalized current-voltage relation of the peak ON responses. Data points represent the average normalized value of the peak current amplitude at each holding potential. The curve indicates that the reversal potential of the ON response was 2.5 mV.

**Figure 3:**
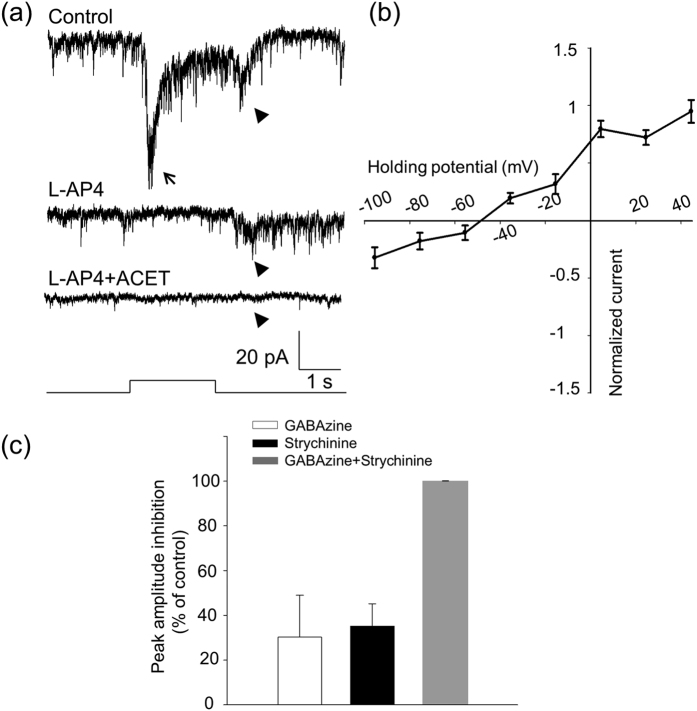
The OFF response of DACs is blocked by ACET and is an inhibitory current. (**a)** A DAC displayed ON (indicated by an arrow) and OFF (indicated by an arrowhead) responses (top trace). The ON response was blocked by L-AP4 (middle trace), whereas the OFF response was eliminated by ACET (bottom trace). Stimulation bar under each trace shows timing of a 470-nm light pulse. Light stimulus duration was 2 s. **(b)** An I–V curve of the OFF responses shows that the reversal potential was −48.5 mV (n = 9). **(c)** GABAzine (white bar, n = 10) and strychnine (black bar, n = 8) each partially suppressed the peak amplitude of the OFF response, whereas their co-application completely blocked the response (grey bar, n = 8).

**Figure 4:**
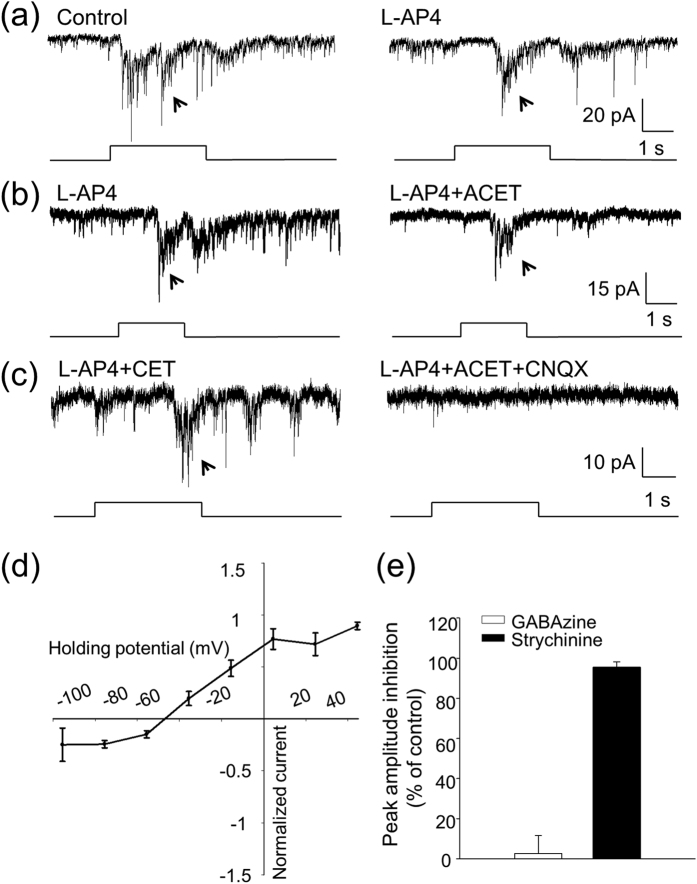
Pharmacological and biophysical properties of d-ON responses of DACs. (**a)** A d-ON response (indicated by an arrow, left trace) was observed in a DAC, and was persistent in the presence of L-AP4 (right trace). **(b)** An L-AP4-resistant d-ON response (arrow, left trace) was not blocked by ACET (right trace) in another DAC. **(c)** An L-AP4 and ACET-resistant d-ON response (arrow, left trace) was completely blocked by CNQX (right trace) in this DAC. Stimulation bar under each trace shows timing of a 470-nm light pulse. Light stimulus duration was 3 s for (**a**) and (**c**) and 2 s for (**b**). **(d)** Normalized current–voltage relation of the peak d-ON responses. Data points represent average normalized values of the peak current amplitude at each voltage. The curve indicates that the reversal potential of the ON response was −46.5 mV. **(e)** GABAzine had almost no effect on the peak amplitude of the d-ON response (white bar, n = 11), whereas strychnine reduced the peak amplitude of the d-ON response by approximately 96% (black bar, n = 8).

Unless indicated otherwise, experiments were conducted using cone-function-only mice in which DACs were genetically labelled with red fluorescent protein (RFP) under the control of the *tyrosine hydroxylase (TH)* promoter. Whole-cell voltage-clamp recordings were made from RFP-positive DACs in flat-mount retinas^34^,^35^. After a whole-cell recording was made, the cell membrane potential was held at −65.5 mV. Then, a 470-nm light flash with an intensity of 2.97 × 10^11^ photons·s^−1^·cm^−2^ (which is near the saturation intensity of cones) was repeatedly delivered to the retina for 2 or 3 seconds every 2 minutes. Light-evoked postsynaptic inward currents were recorded from 86 cells. The majority of the cells (68.6%, Fig. 1d) showed an inward current at light onset (ON response). The inward currents gradually returned toward the baseline before light offset (Fig. 1a). In addition, 18 out of the 86 cells (20.9%, Fig. 1d) had both the ON response and a transient inward current at light offset (Fig. 1b). The peak amplitude of the OFF currents (13.5 ± 1.8 pA, n = 7) was smaller than that of the ON responses (24.4 ± 2.7 pA, n = 7). We also observed a transient inward current superimposed on the decaying phase of the ON response during light in a small percentage of the cells (10.5%, Fig. 1c,d). The average latency of these inward currents is 1317.2 ± 142.8 ms (n = 6), which is much longer than that of the initial ON responses (143.9 ± 27.3 ms, n = 8). We therefore referred to this current as a delayed ON response (d-ON response). The average peak amplitude of d-ON responses was 10.8 ± 1.6 pA (n = 7). Collectively, the data suggest that cones produce three classes of light-evoked inward currents on DACs when held at −65.5 mV. Next, we show that the ON responses are excitatory postsynaptic currents (Fig. 2), whereas the d-ON (Fig. 3) and OFF responses (Fig. 4) are inhibitory postsynaptic currents.

### The initial ON response is an excitatory inward current

Previous studies have demonstrated that the ON responses of DACs are mediated by ON bipolar cells^14^,^15^. We obtained consistent results with the use of L-AP4, an agonist of mGluR6 receptors that selectively blocks the ON pathway of the retina^19^. Figure 2a illustrates a cell that had only an ON response, which was completely blocked by L-AP4 (n = 5). Figure 2b displays a cell that had both ON and OFF responses. L-AP4 selectively blocked the ON response in this and five other cells. To determine if ON bipolar cells drive DACs via excitatory glutamatergic synapses, we measured the ON responses at holding potentials varying from −100 mV to 40 mV with steps of 20 mV. The current–voltage relationship (Fig. 2c) indicates that the reversal potential of the ON response was about 2.5 mV, close to an excitatory ion reversal potential of 0 mV. This suggests that the ON response is mediated by excitatory glutamatergic input.

### The OFF response is an inhibitory inward current

The OFF response of DACs has been previously observed in the presence of L-AP4^15^,^16^. However, our data showed that the OFF response of DACs was present in the absence of L-AP4 (Fig. 1b). We further found that in the presence of L-AP4, the response was reduced; however, the reduction was not significant (Fig. 2b-bottom trace; control: 14.32 ± 3.37 pA vs. L-AP4: 9.89 ± 2.13 pA, paired t-test, *p* = 0.101, n = 4). It was thus hypothesized that this response is mediated by OFF bipolar cells. To verify this hypothesis, we tested whether ACET, a kainate receptor antagonist reported to specifically block signal transmission from photoreceptors to OFF bipolar cells^36^,^37^, has an effect on the OFF response of DACs. Figure 3a illustrates a typical cell with ON and OFF responses (top trace). We used L-AP4 to block the ON response (Fig. 3a middle trace) and then tested whether adding ACET has an effect on the OFF response. We found that ACET completely abolished the OFF response (Fig. 3a-bottom trace). The same results were obtained for 9 other cells, suggesting that the OFF response is likely mediated by OFF bipolar cells expressing kainate receptors.

To determine whether the OFF response is mediated by a glutamatergic input from OFF bipolar cells, we measured light-evoked OFF responses at various holding potentials. We found that the reversal potential of the OFF response was −48.5 mV (Fig. 3b), which is near the equilibrium potential for the chloride ion (−51.7 mV), indicating that OFF bipolar cells do not drive DACs directly through glutamatergic input, but instead indirectly through inhibitory amacrine cells. This conclusion is further supported by pharmacological evidence. The GABA_A_ receptor blocker GABAzine (20 μM) and the glycine receptor blocker strychnine (3 μM) each partially suppressed the OFF response when applied separately (GABAzine: 30.3 ± 18.7%, n = 10 vs. strychnine: 35.2 ± 9.9%, n = 8), and completely abolished the OFF response when applied concurrently (Fig. 3c). In addition, the GABA_C_ receptor antagonist TPMPA had no effect on the OFF responses (n = 3, data not shown). Taken together, these results lead us to conclude that the OFF response is an inhibitory postsynaptic current.

These results raise the question of whether the cells that had only ON responses at a holding potential of −65.5 mV (Fig. 1a) receive OFF inhibition at different holding potentials. We therefore examined the light-evoked responses of these DACs at a holding potential of 4.5 mV. Among 36 cells, 9 (25%) did not have a detectable response at 4.5 mV, indicating that these ON DACs received either weak OFF inhibition or no OFF inhibition (see Supplementary Fig. S4a). The majority of DACs (27 or 75%) exhibited an outward current at light offset, suggesting that these cells receive OFF inhibition (see Supplementary Fig. S4b).

### The d-ON response is an inhibitory current

The most prominent feature of the d-ON response is that it had over one-second latency from light onset (Fig. 1c). To determine whether the d-ON response is mediated through ON or OFF bipolar cells, we tested the effect of L-AP4 and found that it failed to block the d-ON response (Fig. 4a, n = 3). This suggests that it is unlikely that the d-ON response is mediated by ON bipolar cells. We also tested the effect of ACET to determine if the d-ON response is mediated by OFF bipolar cells expressing the kainate receptor. It was found that the d-ON response persisted in the presence of ACET (Fig. 4b). Average data showed that ACET did not significantly change the peak amplitude of the d-ON response (control: 12.2 ± 5.0 pA vs. ACET: 14.1 ± 5.8 pA, paired-*t* test, *p* = 0.141, n = 4). However, CNQX, an antagonist of AMPA/kainate receptors, completely blocked the ACET-resistant d-ON response (Fig. 4c, n = 9), indicating that the d-ON response could be mediated by an OFF pathway that expresses AMPA-type glutamate receptors^38^,^39^.

The current-voltage relation of the d-ON response indicates that the reversal potential was −46.5 mV, which is near the equilibrium potential for the chloride ion (−51.7 mV; Fig. 4d), suggesting that the d-ON response is mediated solely by inhibitory input. This inhibitory input is likely mediated by glycinergic amacrine cells because strychnine almost completely blocked the d-ON response (inhibition: 95.6% ± 2.5%, n = 4, Fig. 4e), whereas GABAzine had no significant effect (inhibition: 2.7% ± 8.8%, n = 5, Fig. 4e).

We showed that 10.5% of DACs exhibited the d-ON response when held at −65.5 mV (Fig. 1d), suggesting that the majority of the cells do not have a d-ON response at this holding potential. Because the holding potential of −65.5 mV was close to the reversal potential of the d-ON responses, there was a possibility that the d-ON response could be undetected. We therefore tested the light response of these cells at a holding potential of 4.5 mV. Out of 48 cells, 24 (50%) exhibited a d-ON response at 4.5 mV (see Supplementary Fig. S5), suggesting that the d-ON response is undetected in 50% of DACs at a holding potential of −65.5 mV.

Collectively, our data demonstrated that the ON response of DACs is an excitatory current, whereas the d-ON response and OFF response are inhibitory currents. Since functional ON, OFF, and ON-OFF amacrine cells are classified according to the peak excitatory currents at light onset and offset, our data suggest that all DACs are ON-type, with some of them having either OFF inhibition or d-ON and OFF inhibitions. The ON and OFF inhibitions appear to be mediated by distinct OFF bipolar cells and amacrine cells.

Although the gross morphology of the retina was largely unaffected by removing rod and melanopsin function, there was some degeneration with age^40^. To rule out secondary degeneration as a potential cause of the distinct classes of light responses in DACs, we examined light-evoked responses of DACs in retinas isolated from wild-type mice with a mixed C57BL/129 background. Our data showed that all three classes of light responses observed in cone-function-only mice were also seen in wild-type animals (see Supplementary Fig. S6).

### ON bipolar cells drive DACs directly and indirectly via ipRGCs

**Figure 5:**
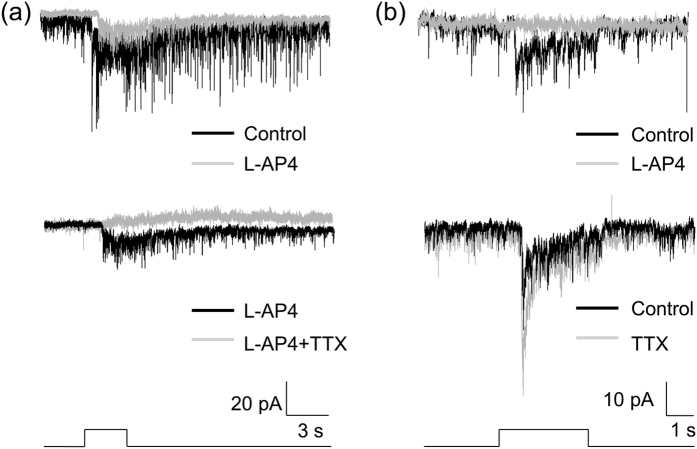
TTX specifically blocks ipRGC input to DACs. Experiments were conducted using wild-type *TH*::RFP mice. **(a)** A typical DAC exhibited an inward current at light onset (top black trace), which was partially blocked by L-AP4 (top grey trace). The L-AP resistant response is mediated by input from ipRGCs. Application of TTX abolished the L-AP4-resistant light response (bottom grey trace), indicating that TTX blocks ipRGC input to DACs. **(b)** A DAC had an inward current at light onset (top black trace), which was completely blocked by L-AP4, suggesting that this current is mediated by ON bipolar cells. After washout of L-AP4 (bottom dark trace), application of TTX increased the light-evoked inward current (bottom grey trace). Stimulation bar under each trace shows timing of a 470-nm light pulse. Light stimulus duration was 3 s.

Cone signals may be sent to DACs by ON bipolar cells directly or indirectly through ipRGCs^16^,^25^,^26^,^27^. Here we attempted to pharmacologically separate the indirect from the direct input using TTX, an action potential blocker. We have previously shown that signal transmission from ipRGCs to DACs is blocked by TTX^41^. Figure 5a illustrates an example of our results, showing that the L-AP4-resistant response of a DAC (ipRGC signalling) was completely blocked by TTX in a wild-type retina. However, it is not known whether TTX can suppress direct input to DACs from ON bipolar cells. Since synaptic input from bipolar cells to DACs is dependent on graded potentials, it is unlikely that TTX blocks this direct input. To rule out such a scenario, we identified 6 cells in wild-type retinas in which light responses were completely blocked by L-AP4 (Fig. 5b-top traces). This suggests that these cells only receive direct input from ON bipolar cells. When TTX was applied, the peak amplitudes of the light response were enhanced in 3 cells (Fig. 5b-bottom traces) and remained unchanged in another 3 cells (data not shown), suggesting that TTX does not block direct input from ON bipolar cells to DACs. In other words, these results and our previous studies suggest that TTX preferentially blocks signal transmission from ipRGCs to DACs in the retina^41^.

**Figure 6:**
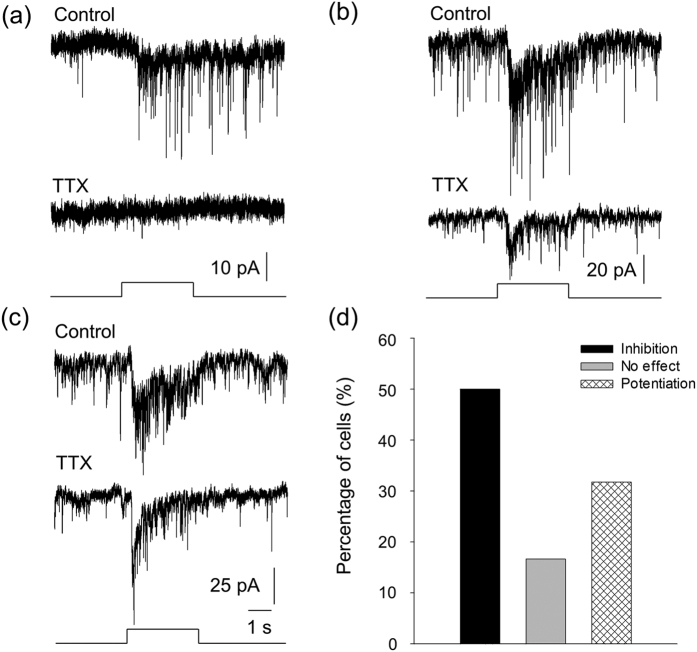
ipRGCs convey cone signals from ON bipolar cells to DACs. Experiments were performed using cone-function-only mice. **(a)** TTX completely blocked the ON response of a DAC, indicating that this cell received input from ON bipolar cells indirectly via ipRGCs (n = 6). **(b)** TTX partially inhibited the peak current amplitude of an ON response of a DAC, indicating that the cell received input from ON bipolar cells both directly and indirectly via ipRGCs (n = 9). **(c)** TTX increased the peak current amplitude of the ON response of a DAC, indicating this cell only receives input directly from ON bipolar cells (n = 12). Stimulation bar under each trace shows timing of a 470-nm light pulse. Light stimulus duration was 3 s. **(d)** Out of 32 cells tested, light-evoked ON responses were either eliminated or suppressed by TTX in 15 cells (black bar), while they were not affected in 5 cells (grey bar) and were enhanced in 12 cells (striped bar).

We then used TTX in cone-function-only mice in which the contribution of melanopsin-based signalling from ipRGCs to DACs is eliminated, and rod function is genetically removed. Any suppression of the light-evoked responses of DACs by TTX in this mouse line should be a result of blocking the cone signal from ipRGCs to DACs. In 15 out of 32 cells tested (46.9%, Fig. 6d), the light responses were either completely blocked (Fig. 6a, n = 6) or partially suppressed (Fig. 6b, n = 9), indicating that these DACs (at least partially) receive input from ON bipolar cells via ipRGCs. For the rest of the cells, TTX either did not have an effect (Fig. 6d, 15.6% of cells, n = 5) or enhanced the peak amplitude of the light responses (Fig. 6c,d, 37.5% of cells, n = 12), suggesting that these DACs receive direct input from ON bipolar cells. Taken together, our data suggest that approximately 50% of DACs receive direct input from ON bipolar cells, while the other half receive additional indirect input from ON bipolar cells via ipRGCs.

### Morphological profiles of ON-type DACs with or without inhibition

**Figure 7:**
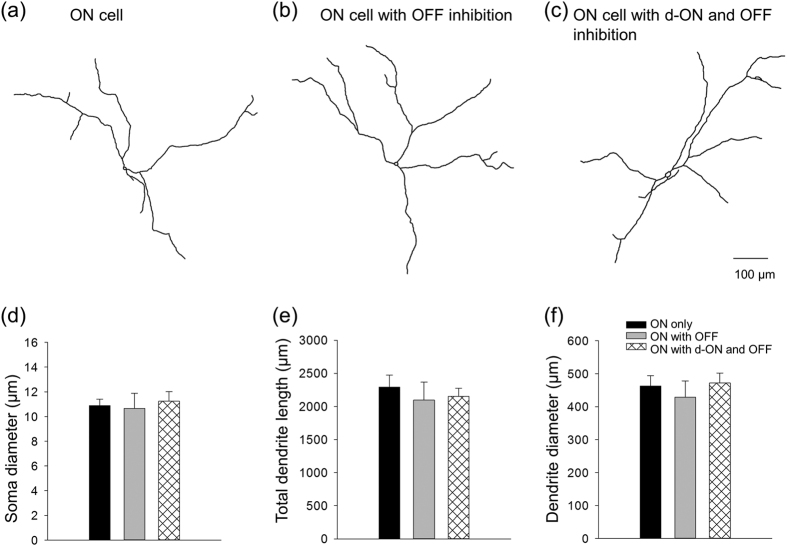
DACs with or without inhibition are of the same morphological type. The morphology of light-responsive cells was revealed by Lucifer Yellow. Drawings of Lucifer Yellow-filled DACs are shown in **(a)**, an ON cell, **(b)**, an ON cell with OFF inhibition, and **(c),** an ON cell with d-ON and OFF inhibition. **(d)** Average data show that there were no significant differences in soma diameter (μm) across ON cells (n = 5), ON cells with OFF inhibition (n = 4), and ON cells with ON and OFF inhibition (n = 7; one-way ANOVA, *p *= 0.879). **(e)** The total dendrite lengths (μm) were also not significantly different (ON cells, n = 4; ON cells with OFF inhibition, n = 3; ON cells with ON and OFF inhibition, n = 6; one-way ANOVA, *p *= 0.768). **(f)** All cells had similar dendrite field diameter (μm) (ON cells, n = 4; ON cells with OFF inhibition, n = 3; and ON cells with ON and OFF inhibition, n = 6; one-way ANOVA, *p *= 0.691).

Since dendrites of RFP^+^ DACs overlap throughout the retina, the morphological profiles of recorded RFP^+^ DACs cannot be distinguished from non-recorded RFP^+^ DACs. To determine whether DACs constitute several morphologically heterogeneous types, we included Lucifer Yellow fluorescent dye in the recording pipette to reveal the morphological features of individual RFP^+^ DACs. Figure 7a depicts the morphology of a typical cell without detectable inhibition (−65.5 mV and 4.5 mV). The cell had a soma diameter of 11.9 μm, which is slightly smaller than reported values^34^. This may be due to the leakage of intracellular content into the cell’s vicinity after the recording pipette was removed from the cell. Four primary dendrites emerged from the soma and branched in a radial manner with a dendrite field diameter of 519.8 μm. Similar features were observed from a typical cell with OFF inhibition (Fig. 7b) and a cell with ON and OFF inhibition (Fig. 7c). Average data showed that there were no significant differences in soma diameter (Fig. 7d), total dendrite lengths (Fig. 7e), and dendrite field diameter (Fig. 7f) across ON cells, ON cells with OFF inhibition, and ON cells with ON and OFF inhibition. These results indicate that ON-type DACs with or without inhibition are likely of the same morphological type.

### Light-induced release of dopamine in cone-function-only mice

To reveal whether cones alone are sufficient to trigger dopamine release through the proposed neural pathways, the ratio of 3,4-dihydroxyphenylacetic acid (DOPAC) to dopamine, an indicator for dopamine release^42^,^43^, in the eyeball was determined in cone-only-function mice. We found that levels of dopamine were slightly increased in light-adapted animals, but this increase was not significant (dark: 926.6 ± 43.1 pg, n = 10 vs. light: 1034.5 ± 49.8 pg, n = 12, Student-*t* test, *p *= 0.125). However, the content of DOPAC was significantly increased in light-adapted animals (dark: 261.3 ± 7.9 pg, n = 10 vs. light: 370.9 ± 20.7 pg, n = 12, student-*t* test, *p *= 0.0002). As a result, the ratio of DOPAC to dopamine was significantly increased (dark: 0.28 ± 0.01, n = 10, vs. light: 0.33 ± 0.02, n = 12, student-*t* test, *p *= 0.018), suggesting that the signalling input of cones to DACs is sufficient to trigger dopamine release.

## Discussion

We have demonstrated that (1) cones alone generate three classes of light responses in DACs: an excitatory ON response, a delayed inhibitory ON response, and an inhibitory OFF response; (2) the excitatory ON responses are mediated by ON bipolar cells both directly and indirectly, via ipRGCs; (3) the inhibitory ON response is mediated by glycinergic amacrine cells that are likely driven by OFF bipolar cells expressing the AMPA receptor; (4) the inhibitory OFF response is mediated by GABAergic/glycinergic amacrine cells that appear to be driven by OFF bipolar cells expressing the kainate receptor; (5) DACs with distinct light responses have indistinguishable morphological profiles; and (6) dopamine release is triggered by light in the cone-function-only eye. Our results suggest that cones utilize multiple neural pathways that convey excitatory and inhibitory signals in parallel to DACs, enhancing retinal dopamine release according to the prevailing illumination.

Previous studies have demonstrated that DACs are driven by rod and cone photoreceptors via ON bipolar cells, exhibiting an ON response in the wild-type retina^14^,^15^,^17^. Here, we expanded previous studies by providing detailed neural circuit mechanisms for the ON response. First, our data suggest that cones alone are sufficient for generating the ON response in DACs, which proves the previous proposal that cones are involved in facilitating DAC activity^12^,^13^. The data in the present study were collected from the cone-function-only mouse model, which allows us to distinguish cone signals from rod signals in DACs. To our knowledge, this mouse model is the only way to accomplish our experimental goals, as the spectral sensitivity of rods greatly overlaps with that of M-cones in mice^22^, and no pharmacological tools are available to separate these two signals.

Secondly, we demonstrated that the reversal potential of the ON response is near the excitatory ion reversal potential. This new evidence shows that the synaptic input from ON bipolar cells is glutamatergic. Previous pharmacological studies have demonstrated that the ON response is blocked by an AMPA/kainate receptor antagonist and is resistant to GABA/glycine receptor antagonists^14^. Combined with our current data, we can conclude that ON bipolar cells provide excitatory synaptic input to DACs, which depolarizes the DACs and potentially triggers dopamine release. Finally, our TTX results suggest that ON bipolar cells excite DACs through direct and indirect pathways (Fig. 6). The current results (Fig. 5) and those from our previous studies^41^ confirm that TTX selectively suppresses signal transmission from ipRGCs to DACs. Using this pharmacological tool in combination with the cone-function-only mouse model, we found that the cone-mediated responses from 50% of DACs were either unaffected or potentiated by TTX. This result indicates that these cells receive cone signals directly from ON bipolar cells. This presumption is supported by previous anatomical studies showing that conventional ON bipolar cells could make contacts with the DAC processes (if any) in the sublamina a (ON layer) of the IPL^14^,^44^ and/or ectopic ON bipolar cell synapses onto DACs in the OFF layer in the mouse retina^16^.

In the other 50% of DACs, however, TTX profoundly suppressed cone-mediated responses in DACs, suggesting that in addition to the direct input referenced above, ON bipolar cells send cone signals indirectly to DACs via ipRGCs. This assumption is supported by previous anatomical studies demonstrating that ON bipolar cells make ectopic synapses with ipRGCs in the OFF layer of the IPL^16^,^25^ and that ipRGCs make putative contacts with DACs via their axon collaterals^41^. Therefore, the present study provides the first evidence demonstrating that ipRGCs play an important role in sending cone signals from the innermost retina back to the outer retina through the dopaminergic system.

Our results also showed that cones generate inhibitory ON and OFF responses in DACs through distinct neural pathways. A line of evidence suggests that the d-ON response and the OFF response are both mediated by the OFF pathway. First, both responses are resistant to L-AP4, indicating that they are not mediated by the ON pathway. Secondly, the d-ON response is always accompanied by the OFF response, suggesting that a shared OFF pathway initiates both responses. Finally, both responses are mediated by ionotropic glutamate receptors, a subtype of glutamate receptor that is expressed on OFF bipolar cells but not on ON bipolar cells. In addition, our results further demonstrated that both the d-ON response and the OFF response do not represent direct glutamatergic input from OFF bipolar cells, even though the dendrites of DACs are primarily located in the OFF layer^12^; they are instead triggered by inhibitory input from other inhibitory amacrine cells. The difference between them is that the former is mediated exclusively by glycinergic amacrine cells, whereas the latter is mediated by glycinergic and GABAergic input.

**Figure 8:**
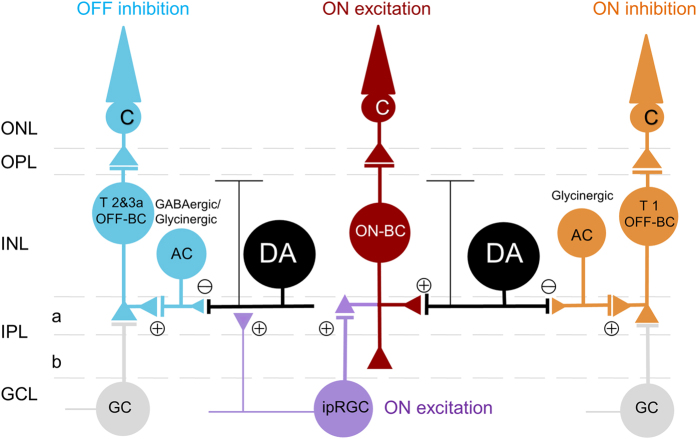
Proposed neural pathways responsible for conveying cone signals to DACs. Excitatory ON responses are mediated by ON bipolar cells directly (red) and indirectly via ipRGCs (violet). OFF inhibition is mediated by type 2 and 3a OFF bipolar cells through GABAergic/glycinergic amacrine cells (cyan), whereas ON inhibition (d-ON response) is mediated by type 1 OFF bipolar cells via glycinergic amacrine cells (orange). GC: ganglion cell; ONL: outer nuclear layer; OPL: outer plexiform layer; INL: inner nuclear layer; IPL: inner plexiform layer; GCL: ganglion cell layer; a: sublamina a/OFF layer; b: sublamina b/ON layer.

Therefore, our proposed neural pathway for ON inhibition would be as follows: cone → OFF bipolar cell → glycinergic amacrine cell → DAC (Fig. 8). Our data further demonstrated that this pathway is exclusively mediated by AMPA receptors. In this pathway, glycinergic amacrine cells co-express AMPA and kainate receptors^45^, while one subtype of OFF bipolar cells, type 1 OFF bipolar cells, exclusively express AMPA-type glutamate receptors^38^,^39^. We thus argue that the selective mediation of the ON inhibition by AMPA receptors is likely initiated on type 1 OFF bipolar cells. That is, type 1 OFF bipolar cells transmit cone signals to glycinergic amacrine cells, which send a crossover inhibitory signal to DACs to generate an ON inhibition at light onset^46^.

In contrast, our data revealed that the neural pathway for the OFF response is solely mediated by kainate receptors. This specific mediation by kainate receptors is not likely to occur in glycinergic/GABAergic amacrine cells, as they co-express kainate and AMPA receptors^45^. Instead, type 2 and 3a OFF bipolar cells that only express kainate receptors^36^,^37^,^38^,^39^ could initiate the OFF response in response to glutamate release from cones at light offset. Therefore, our proposed neural circuit for the OFF inhibition would be as follows: cone → type 2/3a OFF bipolar cell → GABAergic/glycinergic amacrine cell → DAC (Fig. 8). Although further confirmation is needed, the data suggest that OFF bipolar cells could use distinct non-NMDA glutamate receptors to mediate ON and OFF inhibition at the very first synapse of the retina.

We also noted that the latency of the d-ON inhibition was more than 1 second, which is likely why this response was not observed in previous studies that used short light pulses^14^,^15^. Interestingly, the latency of DAC ON inhibition is similar to that of the excitatory ON responses of the OFF pathway in retinal ganglion cells (1.14 s in the mGluR6 knockout retina)^47^. However, the latter was unmasked by L-AP4 and was resistant to inhibitory receptor antagonists. Therefore, we speculate that the generation of the d-ON inhibition of DACs shares OFF bipolar cells with the long-latency excitatory ON response of retinal ganglion cells. The difference could be that this OFF bipolar cell subtype sends glutamatergic input to retinal ganglion cells directly but indirectly to DACs via a glycinergic amacrine cell.

Although some DACs receive OFF inhibition and ON inhibition, their morphological profiles are similar to those of DACs without inhibition (Fig. 7). Notably, we did not attempt to measure the axon-like processes of DACs because they are not completely filled by Lucifer Yellow injection. However, synaptic input to a cell is determined by its dendritic field and dendritic arborization. Consequentially, the lack of morphological data from DAC axon-like processes is unlikely to compromise our conclusion that DACs with or without inhibitory input are morphologically similar. Since 50% of DACs receive cone input from ipRGCs, the cells injected with dye almost certainly include ipRGC-driven DACs. In the wild-type retina, these DACs also receive melanopsin-based signalling from ipRGCs and exhibit a sluggish ON response^17^. We postulated that these ipRGC-driven DACs likely have the same morphology as L-AP4-sensitive cone-driven DACs. Previous studies have demonstrated that ipRGC-driven DACs are predominantly located in the dorsal retina^48^. Since we randomly selected RFP-labelled DACs for recording, cells recorded in the ventral retina may not have received ipRGC input, which gave the appearance that only 50% of DACs exhibited ipRGC signalling.

Finally, our data show that cones alone are sufficient to trigger dopamine release. The main driving force for dopamine release is postulated to be direct excitatory input from ON bipolar cells and indirect input through ipRGCs to DACs. These excitatory inputs could be modulated by ON and OFF inhibition. For instance, when DACs are depolarized by light, the membrane depolarization results in an increased inhibitory input and negatively regulates the excitatory input. Therefore, ON and OFF inhibitions could play a feedback role in regulating DAC activity during illumination and light offset, respectively. Overall, balanced excitatory and inhibitory inputs to DACs are essential for mediating dopamine release in light and dark adapted conditions.

## Materials and Methods

### Animals

Adult female and male mice were used in the experiments. The animals were housed in the Oakland University animal facility on a 12:12-h light-dark cycle, with lights on at 07.30 h. Food and water were available *ad libitum.* All procedures conformed to NIH guidelines for laboratory animals and were approved by the Institutional Animal Care and Use Committee at Oakland University.

The *TH*::RFP mouse line was originally created on a C57BL/6J background at Vanderbilt University^34^ and was imported to Oakland University for the present study. The mice were either used for the experiment conducted in Fig. 5 or were crossed with a triple-knockout mouse line (BL6/129) in which cone photoreceptor-specific cyclic nucleotide channel *Cnga3*, rod specific-G protein transducin α-subunit *Gnat1*, and melanopsin *Opn4* were deleted^28^. From multiple crossings, *TH*::RFP transgenic mice homozygous for the *Gnat1* and *Opn4* mutations (*Opn4*^−*/*−^ *Gnat1*^−*/*−^ *TH*::RFP) were bred on a mixed C57BL/129 background. These mice were used for the majority of the experiments. *TH*::RFP transgenic mice (mixed C57BL/129 background) with wild-type *Gnat1*, *Cnga3*, and *Opn4* genes were used for the control experiments in Fig. 5.

### Electrophysiology recording

All experiments used a flat-mount retina preparation and were conducted during the day to avoid a circadian effect. Mice were dark adapted for 1–2 h prior to experiments and then euthanized by CO_2_ overdose and cervical dislocation. Eyes were enucleated under infrared illumination and transferred to a petri dish filled with oxygenated extracellular solution containing (in mM) 125 NaCl, 2.5 KCl, 1 MgSO_4_, 2 CaCl_2_, 1.25 NaHPO_4_, 20 glucose, and 26 NaHCO_3_. Under a dim red light, the cornea and lens were removed from the eyeballs, and the retina was separated from the sclera. The retina was then placed with the photoreceptor side down in a recording chamber mounted on the stage of an upright conventional fluorescence microscope (BX51WI, Olympus, Tokyo, Japan). Oxygenated extracellular medium (pH 7.4 bubbled with 95% O_2_–5% CO_2_) continuously perfused the recording chamber at a rate of 2–3 ml/min, and the superfusate was maintained at 32–34 °C by a temperature control unit (TC-344B, Warner Instruments, Hamden, CT).

The retina was maintained in darkness for approximately 1 hour prior to recording. Cells and recording pipettes were viewed on a computer monitor coupled to a digital camera (XM10, Olympus, Tokyo, Japan) mounted on the microscope. *TH*::RFP-expressing cells were randomly selected throughout the retina after being visualized by fluorescence using a rhodamine filter set. A peak wavelength of 535 nm from a fluorescence LED illumination system (pE-2, CoolLED Ltd., Andover, UK) was used to give a brief “snap-shot” of fluorescence excitation light (1–2 s). For patch-clamp recordings, the identified cells and glass electrodes were visualized using infrared differential interference contrast (IR-DIC) optics (900 nm Nomarski DIC, Olympus, Tokyo, Japan). Experiments began 10–15 min after the cells were located using fluorescence, which allowed the retina to recover from photobleaching (caused by the brief fluorescence excitation light). Recovery may be incomplete during this short dark-adaptation period, so our experiments were likely performed in a partially light-adapted state.

Whole-cell voltage-clamp recordings were made from the soma of RFP-labelled DACs with 4–8 MΩ electrodes, and signals were amplified with an Axopatch 200B amplifier (Molecular Devices, Sunnyvale, CA). The intracellular solution for the whole cell voltage-clamp experiments contained (in mM) 120 Cs-methane sulfonate, 5 EGTA, 10 HEPES, 5 CsCl, 5 NaCl, 0.5 CaCl_2_, 4 Na-ATP, 0.3 Na-GTP, and 5 lidocaine n-ethyl-chloride (QX-314). QX-314 was used for all DAC recordings to improve the space clamp quality of the voltage-clamp and to prevent intrinsic generation of action potentials in cells^41^. The liquid junction potential was −4.5 mV, which was corrected offline. The pH of the intracellular solution was titrated to 7.2–7.4 with CsOH. All electrophysiological data were acquired using a Digidata 1550A digitizer (Molecular Devices, Sunnyvale, CA).

Tetrodotoxin (TTX) and 6-cyano-7-nitroquinoxaline-2,3-dione (CNQX) were purchased from Sigma-Aldrich (St. Louis, MO). L-2-amino-4-phosphonobutyric acid (L-AP4), (*S*)-1-(2-Amino-2-carboxyethyl)-3-(2-carboxy-5-phenylthiophene-3-yl-methyl)-5-methylpyrimidine-2,4-dione (ACET), strychnine, 2-(3-Carboxypropyl)-3-amino-6-(4 methoxyphenyl) pyridazinium bromide (GABAzine), and (1,2,5,6-Tetrahydropyridin-4-yl) methylphosphinic acid (TPMPA) were purchased from R&D Systems (Minneapolis, MN). All drugs were prepared as concentrated stock solutions that were diluted to working concentrations with extracellular medium prior to experiments.

### Light stimulation

Light stimuli were generated using an LED illumination system (pE-2, CoolLED Ltd., Andover, UK). A light pulse of 470-nm wavelength was delivered to the retina through a microscope objective lens (60x). An LED control pad was used to drive the LED. Light intensity was measured at the retinal surface using an optical power meter (units converted from μW/cm^2^ to photons·s^−1^·cm^−2^; model 843-R, Newport, Irvine, CA). A light intensity of 2.97 × 10^11^ photons·s^−1^·cm^−2^ was used for all experiments.

### Immunohistochemistry and morphological analysis

To reveal the morphology of the recorded cells, Lucifer Yellow was added to the intracellular solution with a concentration of 0.1%. After recording, the retina was fixed for 1 h in phosphate buffered saline (PBS, 0.1 M) containing 4% paraformaldehyde. The retina was then blocked with 1% BSA and 0.3% Triton X-100 in PBS for 2 h at room temperature. After blocking, the retina was incubated with a rabbit anti-Lucifer Yellow antibody (1:500; Life Technologies, Grand Island, NY) at 4 °C for 3–5 days, rinsed with PBS, and treated with a secondary antibody conjugated with Alexa-488 (Invitrogen) at room temperature for 2 h. Samples were coverslipped using VECTASHIELD® (Vector Laboratories Inc., Burlingame, CA).

Images were taken at 40x magnification using a Zeiss Axio Imager 2 fluorescence microscope (AX10, Zeiss, Oberkochen, Germany). The MosaiX function was used to obtain tile scan images of the entire retina. NIS Elements AR software was then used to stitch tile images into a single large image. The dendrites of cells injected with Lucifer Yellow were traced manually using the Simple Neurite Tracer plugin for ImageJ (National Institutes of Health; Bethesda, MD). Total dendritic length was measured using the dendrite traces, and dendritic field size was measured by connecting all dendritic tips in a 2-D image with straight lines and calculating the polygon area. Both dendritic field size and soma sizes were calculated as the diameter of a circle of equal area.

### Dopamine and DOPAC measurement

To determine the DOPAC and dopamine content in the eye, the eyeballs were enucleated, rapidly frozen in liquid nitrogen, and kept at −80 °C until assayed. For the dark-adapted group, mice were maintained in the dark from lights off (19.30 h) until the next day (12.00 h) and then euthanized. For the light-adapted group, mice were euthanized on the day of the experiment 4.5 h after lights on (12.00 h). This time point was within the period, typically 10.00 h–17.00 h, when we conducted electrophysiological recordings from DACs. Frozen eyeballs were homogenized by sonication in 200 μL of a solution containing 0.4 M perchloric acid, 0.1 mM Na_2_S_2_O_5_, and 0.1 mM Na_2_-EDTA. The homogenate was then centrifuged (30 min, 16,000 g, 4 °C), and the supernatant was passed through a filter with 0.2-μm pore size. For each sample, 5 μL of supernatant was directly injected into the Reversed-phase high-performance liquid chromatography (HPLC) system.

HPLC with electrochemical detection (ED) was used to assay dopamine and its metabolite DOPAC. The liquid chromatograph and detection system consisted of a Prominence HPLC (Shimadzu Scientific Instruments, Columbia, MD) with a Hypersil™ ODS C18 column (250 mm × 4.6 mm, 5-μm porous silica, ThermoScientific, Grand Island, NY, USA) and an amperometric detector (Model LC-4C, BioAnalytical System, West Lafayette, IN, USA). We used an isocratic mobile phase composed of 50 mM KH_2_PO_4_, 0.015% octyl sodium sulphate (ACROS Organics, NJ, USA), and 0.1 mM Na_2_-EDTA, which was adjusted to pH 3.10 before adding 13% methanol. The solution was then filtered on a membrane filter (pore size 0.2 μm) and degassed with helium. The mobile phase flow rate was set to 1 ml. min^−1^, and the column temperature was set to 30 °C. The potential of the working carbon electrode was set to +600 mV. The analysed substances were identified by their relative retention times compared to those of standards and were quantified based on the peak area. The detection threshold of the HPLC system was 5 pg per run, which was determined with a standard solution.

### Data analyses

Electrophysiological data were analysed offline using Clampfit 10.4 (Molecular Devices, Sunnyvale, CA) and SigmaPlot 12.0 (Systat Software, Germany) software packages. The current of an individual DAC was measured as the peak amplitude at either light onset or offset. To make the current-voltage curves, each cell’s currents were normalized by dividing the peak current amplitude at each holding potential by the maximum peak current for that cell. Normalized peak currents from different cells at the same holding potential were then averaged and plotted against the holding potential. To assess the effects of drugs, the significance of the change in the light-induced current amplitude was determined using a paired t-test. Two independent groups with normally distributed data and unequal sample sizes were compared using a Student t-test. One-way analysis of variance (ANOVA) was used to determine whether there were any significant differences between the means of three independent groups. *p* < 0.05 was considered statistically significant. Data are presented as the mean ± SEM.

## Additional Information

**How to cite this article**: Qiao, S.-N. *et al.* Multiple cone pathways are involved in photic regulation of retinal dopamine. *Sci. Rep.* **6**, 28916; doi: 10.1038/srep28916 (2016).

## Supplementary Material

Supplementary Information
