# Supplementary material for: Multiple cone pathways are involved in photic regulation of retinal dopamine
Source: Sci Rep. 2016 Jun 30;6:28916. doi: 10.1038/srep28916 (PMC4928117; doi:10.1038/srep28916)
Supplement: Supplementary Information [file srep28916-s1.pdf]

Supplementary Information

Multiple cone pathways are involved in photic regulation of retinal dopamine

Sheng-Nan Qiao[1,2], Zhijing Zhang[3], Christophe P. Ribelayga[3,4], Yong-Mei Zhong[1,5,6*], and Dao-Qi Zhang[2*]

[1]*Institutes of Brain Science, Fudan University, Shanghai 200032, China*

[2]*Eye Research Institute, Oakland University, Rochester, MI 48309, USA*

[3]*Ruiz Department of Ophthalmology and Visual Science, The University of Texas Health Science Center at Houston, Houston, TX 77030, USA*

[4]*Graduate School of Biomedical Sciences, The University of Texas Health Science Center at Houston, Houston, TX 77030, USA*

[5]*State Key Laboratory of Medical Neurobiology, Fudan University, Shanghai 200032, China;*

[6]*Collaborative Innovation Center for Brain Science, Fudan University, Shanghai 200032, China*

**Co-corresponding author: zhang@oakland.edu or ymzhong@fudan.edu.cn*

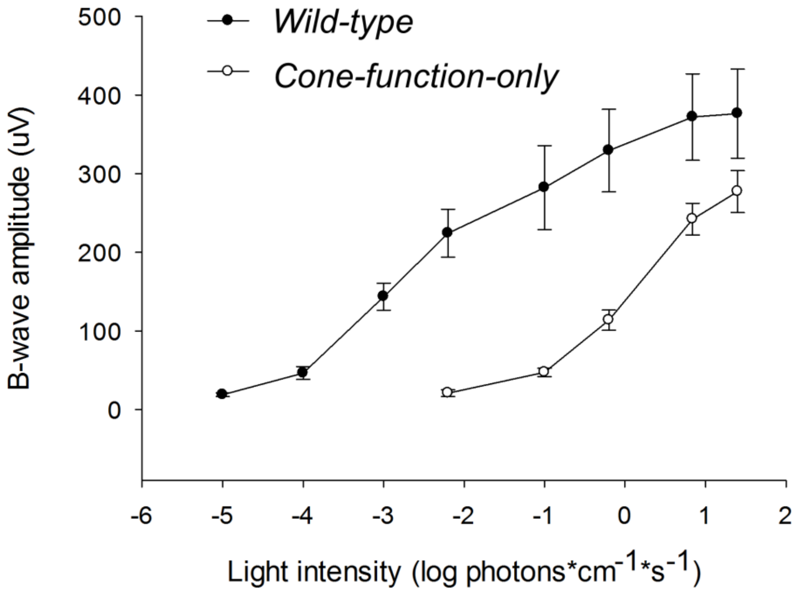

Figure S1. Loss of rod function is evidenced by reduced sensitivity of dark-adapted electroretinogram (ERG) b-wave in cone-function-only mice.

Mouse ERGs were recorded using an Espion-III ERG system and software (Diagnosys LLC, Lowell, MA). After 1 hour of dark adaptation, mouse pupils were dilated by sequential

application of 1% tropicamide and 2.5% phenylephrine eye drops. Anesthesia was induced by a single intraperitoneal injection of 50 mg/kg ketamine HCl and 7 mg/kg xylazine. A sequence of 5 ms white light flashes were given to evoke the ERG at intensities ranging from -5 log photons/cm^2·s to 1.4 log photons/cm^2·s. The b-wave amplitude was measured from the maximum a-wave peak to the maximum b-wave peak. The intensity–response curve of the mean b-wave amplitudes of 3 wild-type mice (closed circles) and 3 cone-function-only mice (open circles) are shown. The intensity-response curve of cone-function-only mice was shifted to the right by more than 3 log units, indicating that rod function is lost in this mouse line.

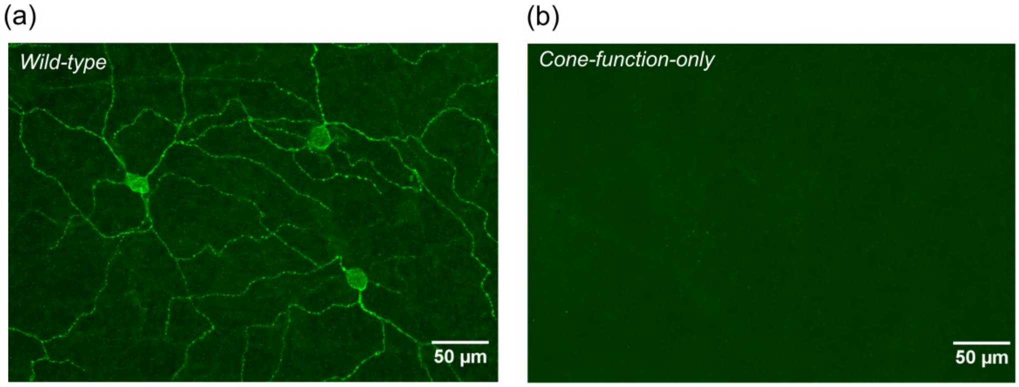

Figure S2. No melanopsin immunostaining was detected in retinas of cone-function-only mice.

Whole retinas were blocked for two hours with 1% bovine serum albumin (Fisher Scientific, Hampton, NH) and 0.3% Triton-X 100 (Sigma, St. Louis, MO) in 0.1x PBS. Retinas were incubated with a primary antibody against melanopsin (1:10,000; AB-N38, Advanced Targeting Systems, San Diego, CA). Retinas were then rinsed in 0.1x PBS and incubated for 2 hours with a secondary antibody raised in donkey, conjugated to AlexaFluor-488 (1:500; Life Technologies; Carlsbad, CA). Finally, whole retinas were mounted on slides with Vectashield Hard-Set mounting solution (Vector Laboratories, Burlingame, CA). Melanopsin-positive cells were observed in a wild-type retina **(a)** but not in a cone-function-only retina (**b**). These experiments were repeated with identical results in 3 wild-type mice and 3 cone-function-only mice.

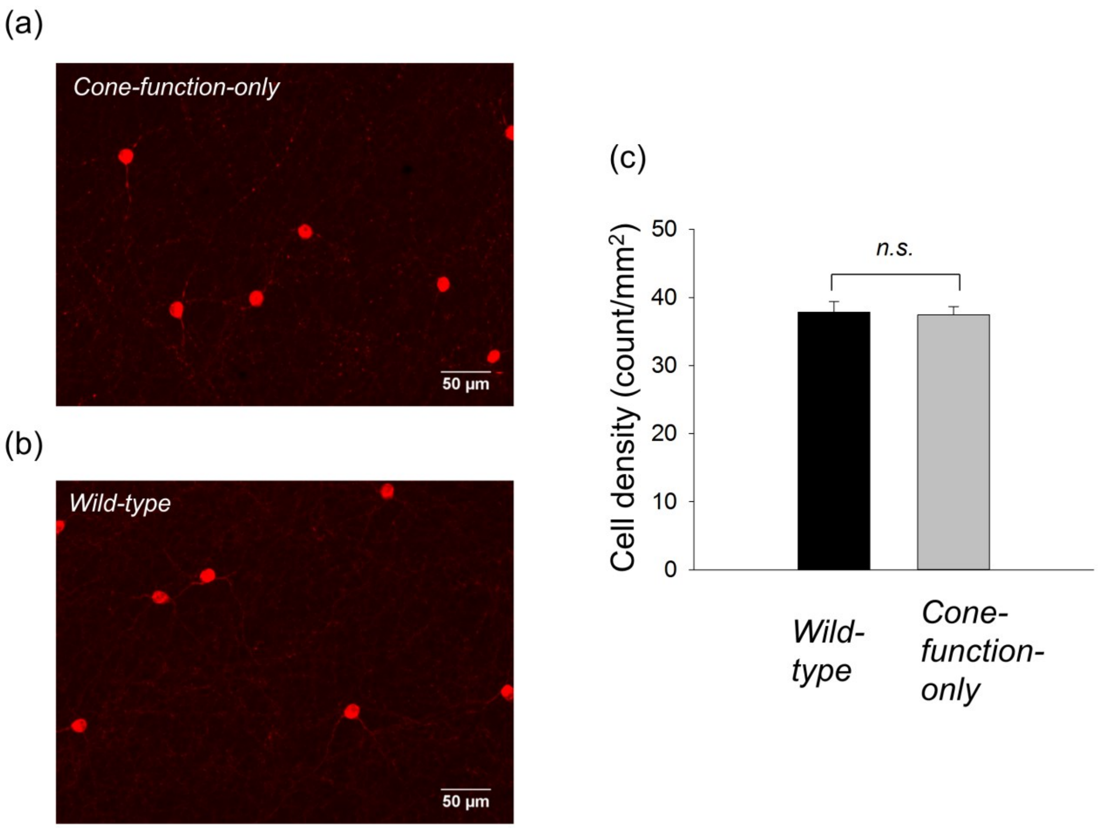

Figure S3. Loss of rod and melanopsin function does not change the number of DACs in the retina.

The same procedures used for melanopsin immunostaining in Figure S2 were used for TH immunostaining in this figure. The primary antibody against TH was used at a concentration of 1:1000 (AB1542, EMD Millipore, Billerica, MA). **(a)**: TH staining in a cone-function-only retina. **(b)**: TH staining in a wild-type retina. **(c)**: The TH-expressing cell density in retinas of wild-type mice (37.9 ± 1.5 /mm^2, n = 3) remained unchanged in retinas of cone-function-only mice (37.5 ± 1.2 /mm^2, n = 3; student *t-test*, P = 0.835).

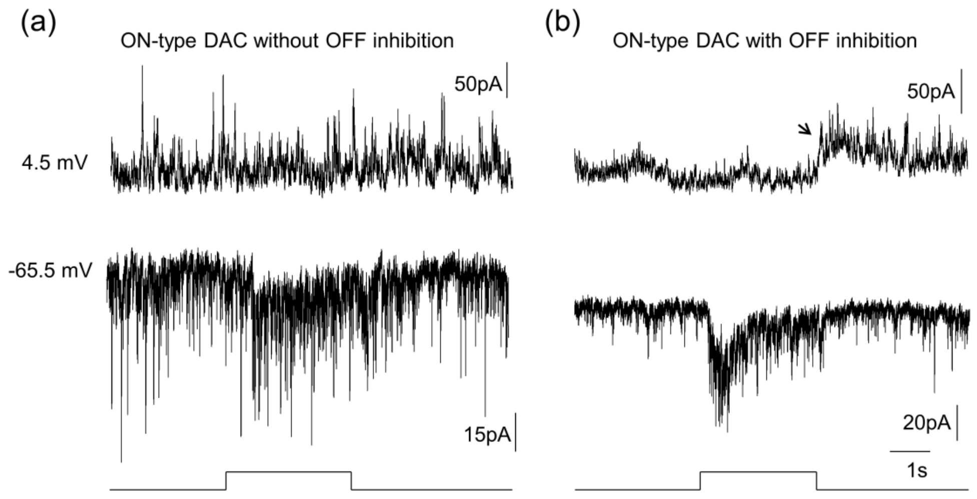

Figure S4. **The majority of DACs that only have excitatory ON responses at -65.6 mV show OFF inhibition at 4.5 mV.**

(a) A DAC exhibited a light-evoked inward current at -65.5 mV but no detectable current at 0 mV, suggesting it is an ON cell. **(b)** A DAC showed an excitatory ON response at -65.5 mV and an inhibitory OFF response at 4.5 mV, indicating this ON cell receives OFF inhibition. Stimulation bar under each panel shows timing of a 470-nm light pulse. Light stimulus duration was 3 s.

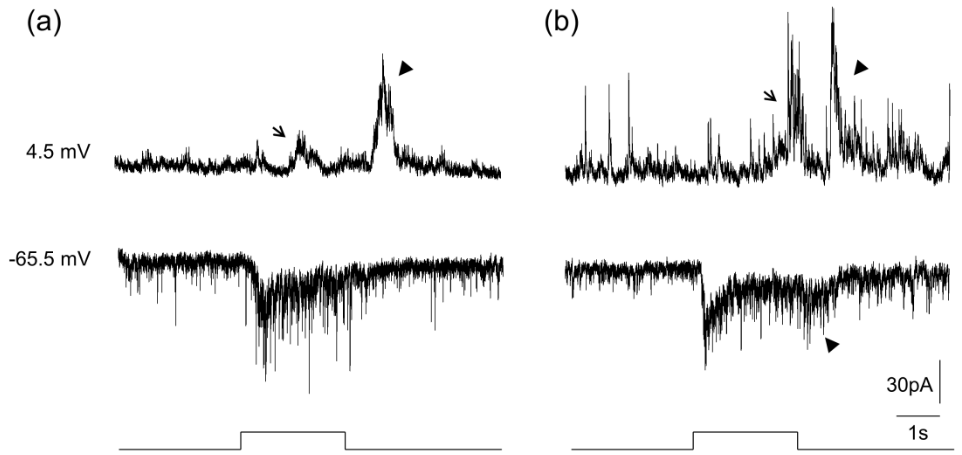

Figure S5. DAC cells without d-ON inhibition at -65.5 mV show d-ON inhibition at 4.5 mV.

(a) A DAC showed an excitatory ON response at -65.5 mV but d-ON (indicated by an arrow) and OFF (indicated by an arrowhead) inhibitions at 4.5 mV, suggesting that this ON cell receives ON and OFF inhibition. **(b)** A DAC that had excitatory ON and inhibitory OFF responses at -65.5 mV exhibited inhibitory ON (indicated by an arrow) and OFF (indicated by an

arrowhead) inhibitions at 4.5 mV. Stimulation bar under each panel shows timing of a 470-nm light pulse. Light stimulus duration was 3 s.

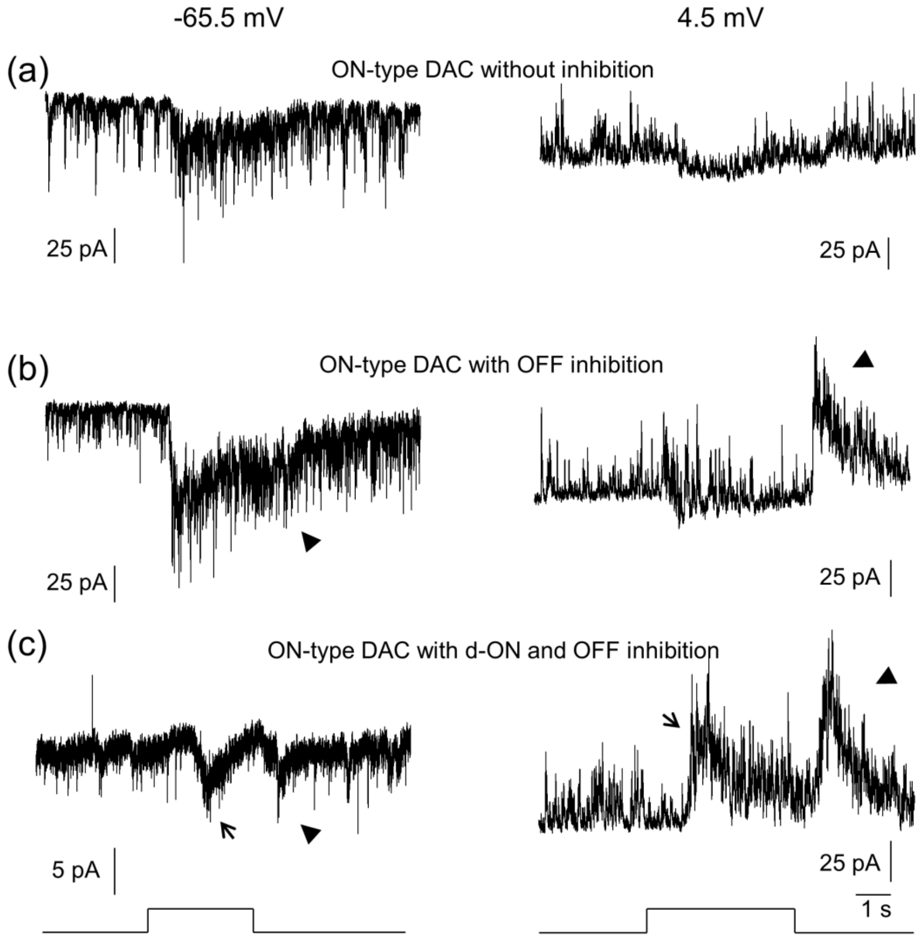

Figure S6. Three classes of light responses of DACs recorded in *wild-type* mice. Voltage-clamp recordings were made from DACs in retinas of wild-type mice with a mixed C57BL/129 background at the holding potentials of -65.5 mV and 4.5 mV, respectively. **(a)** A DAC displayed a relatively large inward current at -65.5 mV (left trace) and a small amplitude inward current at 4.5 mV (right trace) at light onset (n = 3). **(b)** A DAC exhibited ON and OFF (indicated by an arrowhead) responses at both -65.5 mV (left trace) and 4.5 mV (right trace) (n = 4). **(c)** d-ON (indicated by arrows) and OFF (indicated by arrowheads) responses were observed in a DAC at both -65.5 mV (left trace) and 0 mV (right trace) in the presence of TTX and L-AP4. Stimulation bar under each panel shows timing of a 470-nm light pulse. Light stimulus duration was 3 s for traces at -65.5 mV and 4 s for traces at 4.5 mV.
